# Transient gestational hypertension and pre-eclampsia: Two case reports and literature review on the need for stringent monitoring

**DOI:** 10.4102/safp.v63i1.5236

**Published:** 2021-03-16

**Authors:** Nnabuike C. Ngene, Ghadah Daef

**Affiliations:** 1 Department of Obstetrics and Gynaecology, Faculty of Health Sciences, School of Clinical Medicine, University of the Witwatersrand, Johannesburg, South Africa; 2 Department of Obstetrics and Gynaecology, Leratong Hospital, Krugersdorp, South Africa; 3 Department of Obstetrics and Gynaecology, Klerksdorp Hospital, Klerksdorp, South Africa

**Keywords:** hypertensive disorders of pregnancy, intrauterine foetal death, pre-eclampsia, stringent monitoring, transient gestational hypertension

## Abstract

Transient gestation hypertension is a contributor to adverse pregnancy outcomes particularly when it progresses to pre-eclampsia (PE). This requires frequent monitoring. We illustrate the need for stringent monitoring of gestational hypertension, transient gestational hypertension (TGH) and PE without severe features and conducted a brief rapid review of the literature. Two cases are presented: Firstly, a 25-year-old primigravida at 30 gestational weeks who had an isolated TGH with high blood pressure (BP) of 141/87 mmHg, which was not investigated. Four weeks later, she presented with a BP of 202/128 mmHg, imminent eclampsia and intrauterine foetal death and had an uncomplicated induction of labour and delivered a 1400 g macerated male stillborn. Secondly, a 30-year-old primigravida at 30 gestational weeks who developed PE but her monitoring was compromised initially by inadequate healthcare capacity including unavailability of hospital bed-space for inpatient care and later by poor clinic attendance as a result of poor finances. At 32 gestational weeks, she presented with decreased foetal movement and was diagnosed as haemolysis, elevated liver enzymes, low platelet count (HELLP) syndrome and intrauterine foetal death. She was stabilised, had induction of labour and delivered a 1400 g male macerated stillborn. Thereafter, the need for her to go home to complete the cultural burial rites of her baby and the pressure from her workplace resulted in an inadequate postpartum follow-up care. In conclusion, transient gestational hypertension is associated with adverse maternal and foetal outcomes, including foetal demise. Unavailability of hospital bed-space and poor personal finances interfere with stringent monitoring of hypertensive disorders and can be associated with adverse pregnancy outcomes. Stringent laboratory monitoring in these cases is defined by the authors as testing at least blood levels of serum Creatinine, Haemoglobin concentration, Alanine transaminase and Platelet count (abbreviated as ‘CHAP’) weekly.

## Introduction

Hypertensive disorders of pregnancy (HDP) occur in 5% – 10% of pregnancies and account for 14% of maternal deaths worldwide.^1,2^ The burden of the disease is highest in low- and middle-income countries.^1^ In South Africa for instance, HDP accounts for 18% of maternal deaths^3^ and this is because of high prevalence (9.6% for pre-eclampsia [PE]),^4^ the propensity for the severe forms of the disease^5^ and avoidable factors associated with their management.^6^ In 2018, the International Society for the Study of Hypertension in Pregnancy (ISSHP) categorised HDP into chronic hypertension, PE, which may be de novo or superimposed on chronic hypertension, white coat hypertension, masked hypertension, transient gestational hypertension (TGH) and gestational hypertension.^7^ The ISSHP definition of these categories are recognised in the South African 2019 National guidelines on HDP.^3^

Transient gestational hypertension is the brief occurrence of hypertension (systolic blood pressure [BP] of ≥ 140 mmHg and or diastolic BP of ≥ 90 mmHg) at ≥ 20 gestational weeks, followed by normalisation of BP without treatment.^1,7^ Unfortunately, clinical management of TGH is rarely reported. Transient gestational hypertension progresses to gestational hypertension in 20% of cases and PE in 19% of cases^8^ and may result in maternal and foetal complications such as placental insufficiency. In 25% of cases, gestational hypertension also develops into PE.^7^ Therefore, TGH, gestational hypertension and PE require regular and frequent monitoring. Notably, their outcomes can be dramatic^5^ and there is no single laboratory test or variable that predicts the disease progression and outcomes with certainty.^9,10^ Stringent laboratory monitoring, defined by the authors as testing at least blood levels of serum Creatinine, Haemoglobin concentration, Alanine transaminase (ALT) and Platelet count (**CHAP**) weekly in patients already diagnosed to have gestational hypertension, TGH or PE without severe features, may offer the best pregnancy outcomes. Recent reports indicate that the quantity of multiple maternal vascular malperfusion lesions in the placenta in gestational hypertension and PE may be similar,^11^ and this underscores the ability of gestational hypertension to cause adverse pregnancy outcomes. In this article, we present two case reports to demonstrate the risk of unrecognised and poorly monitored TGH and illustrate the influence of socioeconomic challenges in the monitoring of PE.

## Case 1

A 25-year-old primigravida commenced antenatal care in a primary healthcare clinic (PHC) at eight gestational weeks. During the first antenatal clinic visit, she had the following: BP 129/69 mmHg, pulse rate 61 bpm, weight 85 kg and normal body mass index (BMI). She subsequently had five uneventful antenatal clinic visits and a ‘normal’ foetal structural anomaly ultrasonography at 24 weeks’ gestation.

On the 6th antenatal clinic visit at 30 gestational weeks, she had an isolated BP of 141/87 mmHg (measured on two different occasions 15 min apart as recommended in the 2019 South African guidelines on HDP),^3^ treated with no medication and was referred to the hospital for further assessment. The patient presented to the hospital in the afternoon of the same day with no complaint, BP of 133/63 mmHg that was re-checked on two different occasions, normal spot urine dipstick and symphysio-fundal height (SFH) of 28 cm. Her BPs in the previous antenatal clinic visits were normal (systolic BP < 130 mmHg and diastolic BP < 80 mmHg). No further workup was performed. The patient was discharged home from the hospital and advised to continue antenatal care in the PHC in 4 weeks.

During the next scheduled antenatal clinic visit at 34 gestational weeks, the patient presented with headache and a BP of 202/128 mmHg. She was treated with rapid-acting nifedipine 10 mg orally, methyldopa 500 mg orally and loaded with MgSO_4_ and referred to the hospital for further management as a case of PE with severe features. On arrival at the hospital, the patient had headache, epigastric pain and history further revealed that she had not felt foetal movements for 2 days before presentation. She was also found to have BP of 168/106 mmHg, +1 proteinuria, bilateral pitting pedal oedema, soft abdomen, SFH of 30 cm, no foetal heart sound on auscultation and was not in labour. Ultrasonography showed intrauterine foetal death and anhydramnios. The patient was diagnosed as TGH that has progressed to PE.

The patient was admitted to the obstetric high-care unit, where she received MgSO_4_ for 24 h, rapid-acting nifedipine 10 mg stat, methyldopa 500 mg thrice a day and amlodipine 10 mg once daily. The renal function, full blood count and liver function tests were normal. Labour was induced with oral misoprostol and 14 h following hospital admission, she delivered a 1400 g macerated male stillborn, a placenta that weighed 220 g and retroplacental clot was observed. Post-delivery, the BP was controlled with amlodipine 10 mg daily, and the results of blood investigation remained normal. The patient received grief counselling and was discharged home in satisfactory condition on 3 days after childbirth and had a normal postpartum period. The placental histology confirmed retroplacental haematoma, infarction and high grade foetal vascular malperfusion.

## Case 2

A 27-year-old primigravida commenced antenatal care in a PHC at eight gestational weeks. She had no complaint at booking and her BMI was 50 kg/m^2^ (weight 136 kg). Her subsequent four antenatal care clinic visits were uneventful. The structural anomaly ultrasound scan at 21 gestational weeks was normal. The patient presented to the PHC at 30 gestational weeks, with facial puffiness, bilateral pitting oedema and a BP of 143/103 mmHg. She was commenced on methyldopa and referred to the regional hospital for BP control and further investigation. At the regional hospital, she had a BP of 152/88 mmHg, 4+ proteinuria, normal blood investigations for PE and a normal foetal heart rate. The patient was continued on methyldopa, planned for outpatient care because of unavailability of hospital bed-space. She was booked for an ultrasound scan with a sonologist in the next available space in 2 days’ time (as there were too many patients waiting to access prenatal ultrasonography), and to be followed-up afterwards at the regional hospital. Unfortunately, the patient failed to follow-up because it was economically inconvenient.

At 32 gestational weeks, the patient presented to the PHC with decreased foetal movements of 3 days duration and a cramping lower abdominal pain with no other symptom and was referred to the regional hospital where physical examination revealed bilateral pitting pedal oedema, BP of 195/132 mmHg, 3+ proteinuria and an absent foetal heart sound. Other physical examinations were normal. Rapid-acting antihypertensive therapy (nifedipine) was given to control BP. The patient was admitted to the obstetric high care unit and received MgSO_4_ infusion to prevent eclampsia. The blood investigations showed features of HELLP Syndrome: ALT 220 U/L, aspartate transaminase (AST) 523 U/L, lactate dehydrogenase (LDH) 2075 U/L, platelets 31 × 10^9^/L. The haemoglobin was 12.7 g/dL and obstetric ultrasonography confirmed foetal demise. Ultrasonography of the kidney and liver were normal. These were carried out because early-onset PE (i.e. PE developing before 34 gestational weeks) are usually severe^10^ and in our setting, therefore, ultrasonographic assessment of maternal liver and kidney is usually performed to detect any pathology that may be contributory to the clinical features and to exclude complications of the HDP. Nonetheless, the patient was stabilised, had induction of labour with oral misoprostol and delivered 1400 g male macerated stillborn.

Postpartum, she received counselling and was planned for further inpatient care. On day 2 postpartum, the patient requested to be discharged home to complete the traditional burial rites of her baby. Despite counselling about the need for inpatient care, she signed ‘refusal of in-hospital treatment’ and agreed to return to the hospital the next day but defaulted. Her blood investigation results were serum creatinine 85 *µ*mol/L, urea 3 mmol/L, ALT 108 U/L, AST 119 U/L and LDH 1758 U/L. She was followed-up on an outpatient basis but defaulted clinic visits to attend to responsibilities at her workplace. She made a complete recovery with normal blood results and was discharged from the postnatal clinic on week 7 postpartum. During the last postnatal clinic visit, she had BMI 40.8 kg/m^2^, BP 137/88 mmHg, pulse 89 bpm, serum creatinine 59 *µ*mol/L, haemoglobin 12.6 g/dl, ALT 17 U/L and platelet 283 × 10^9^/L.

## Discussion

The pathogenesis of new-onset hypertension during pregnancy is not well understood^12,13^ but we do know that all categories of HDP have the propensity to progress to PE. Of note, PE causes more adverse perinatal and maternal morbidity and mortality than other categories of HDP. Till date, the pathogenesis of PE has been studied more extensively than those of other categories of HDP. In an attempt to explain the pathogenesis of PE, many theories have been proposed and one of the most popular amongst them is the two-stage theory.^12^ In the first stage of the disease, there is a lack of cytotrophoblastic invasion of the uterine spiral artery and this prevents widening of the lumen of these arteries as seen in normal pregnancy. The lumen therefore remains narrow and causes abnormal blood flow through these arteries and results in vascular malperfusion of the placenta. In the second stage of the disease, the malperfusion in conjunction with maternal susceptibility results in damage to the syncytiotrophoblast, which culminates in excessive release of inflammatory mediators, including anti-angiogenic factors known as soluble fms-like tyrosine kinase-1 (sFlt-1). In the absence of maternal susceptibility, lack of spiral artery remodelling will not cause PE but may result in any other placental mediated diseases, that is, great obstetric syndromes such as foetal growth restriction. Nonetheless, the concentration of the anti-angiogenic factors become higher than the concentration of pro-angoiogenic factors such as placental growth factor (PIGF), which is amongst the seven members in the family of vascular endothelial growth factors (VEGF).^13^ The imbalance between the anti- and pro-angiogenic factors (represented as sFlt-1/PIGF ratio), sFlt-1 and PIGF are biomarkers used in clinical practice for predicting, screening and diagnosing PE^12,14^ and has great potential for predicting postpartum antihypertensive drug requirements.^10^ The sFlt-1 damages the vascular endothelium whose healthy state is usually maintained by VEGF. The damage to the vascular endothelium results in the clinical features of PE.^12,13^ The preceding description applies to early-onset-PE. The understanding is that in late onset-PE, the placenta overgrows its blood supply or becomes old and these cause damage to the syncytiotrophoblast and result in the release of the same type of inflammatory mediators, including sFlt-1.^12,13^ The disease causes lesions in the placenta, but heterogeneity was noticed in many placental histopathological reports.

In a meeting held in September 2014 in Amsterdam, 26 pathologists adopted a standardised guideline, which was published in 2016.^15^ Using the consensus terminology,^15^ the groups of histopathological placental lesions that may be caused by PE or foetal growth restriction are: (1) vascular lesions (such as maldevelopment, malperfusion and loss of integrity) in maternal, foetal or feto-maternal side; (2) immunoinflammatory lesions (including infectious and immune types); and (3) other lesions (for instance, massive perivillous fibrin[oid] deposition otherwise known as maternal floor infarction).^16^ The vascular malperfusion lesions in the maternal placental side is associated with ultrasonographic foetoplacental dopplers such as uterine artery dopplers, and this supports the use of foetal dopplers as a means of assessing placental insufficiency.^16^ Recently, it was reported that both gestational hypertension and PE may manifest similar maternal vascular malperfusion lesions in the placenta.^11^

Unfortunately, there is no clear recommendation in the literature on how TGH should be monitored. It is prudent in the authors’ opinion that TGH should be followed-up and managed as gestational hypertension. Therefore, antenatal clinic visit for foetal and maternal surveillance (including laboratory investigations) should be at short intervals not longer than a week^17^ but determined by maternal and foetal well-being measures such as BP, obstetric ultrasonography and screening for the development of features of PE including proteinuria, signs of imminent eclampsia and deranged laboratory tests results. The first case in the present report demonstrates a failure in recognition and follow-up of TGH.

**FIGURE 1:**
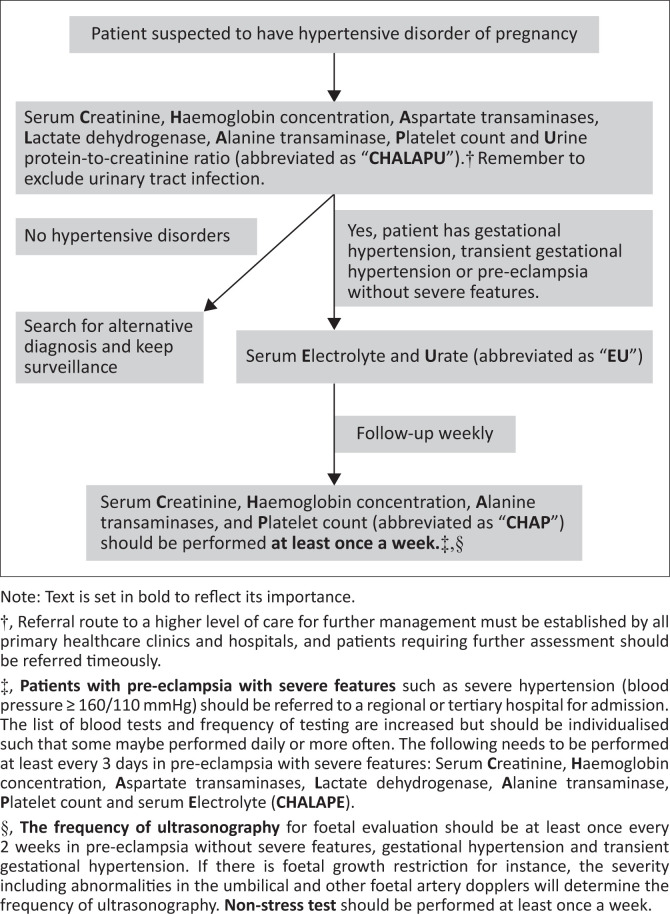
Minimum laboratory tests for a suspected hypertensive disorder of pregnancy and stringent monitoring of gestational hypertension, transient gestational hypertension and pre-eclampsia without severe features.

Pre-eclampsia may also be associated with inadequate monitoring. The National Institute for Health and Care Excellence (NICE) in the United Kingdom recommends that women with PE should have an assessment of full blood count, renal and liver function tests at least twice a week.^17^ Based on expert opinion, the American College of Obstetricians and Gynaecologists (ACOG) recommends that laboratory test for monitoring gestational hypertension and PE without severe features should be performed one to two times weekly.^18^ Because of the latter and given that TGH may progress to PE or gestational hypertension, the laboratory test for monitoring TGH should be carried out at least once weekly. In low resource settings, this schedule is difficult to comply with because of financial constraints and poor educational enlightenment, poor access to healthcare services, poorly skilled healthcare providers and inefficient referral pathways. In South Africa, the 2019 guidelines on HDP recommend that gestational hypertension should be followed-up weekly in the antenatal clinic after initial evaluation with serum creatinine, haemoglobin concentration, ALT, platelet counts and ultrasonography for foetal evaluation to excluded PE.^3^ Unfortunately, the follow-up laboratory tests and frequency of the testing in gestational hypertension and PE are not clearly stated in the same guidelines. However, the South Africa maternity care guidelines recommend less stringent monitoring of weekly platelet and twice-weekly cardiotocography in PE.^19^ Despite the controversies about the ‘ideal’ list of investigations for PE,^20^ the authors’ suggest that at least serum creatinine, haemoglobin concentration, AST, LDH, ALT, platelets and urine protein:creatinine ratio (CHALAPU) should be performed when the diagnosis of gestational hypertension, TGH or PE is being made or excluded. Where available, Angiogenic factors (ratio of sFlt-1/PIGF) may be used to diagnose PE if the clinical features are uncertain^12^ and the mnemonic ‘A-CHALAPU’ instead of ‘CHALAPU’ may be used to remember the necessary laboratory investigations listed here. Serum electrolyte and urate (EU) should then be assessed in patients diagnosed to have PE. Subsequently, serum CHAP should be performed at least once a week in gestational hypertension, TGH and PE without severe features. See Figure 1 for a schematic flow diagram of the recommended laboratory investigations. *The minimum basic set of laboratory investigations that we have recommended are informed by the current criteria used in the definition of PE*^7^ *and laboratory markers that predict poor pregnancy outcomes in PE such as urate, serum creatinine, platelet count and AST.*^21,22,23^ Where more than one laboratory test can identify a complication, we have chosen a single test, for example, the choice of LDH over bilirubin to identify haemolysis resulting from HELLP syndrome. Our recommendations are pragmatic, cost-saving in resource-limited settings and are supported by recent evidence from Canada where Thompson and colleagues in 2020 reported original research findings affirming that basic blood tests required to monitor PE without severe features are complete blood count, ALT and serum creatinine.^24^ Our recommendation is not a disregard for other rare derangements including hypokalaemia that may occur in PE.^25,26^ Generally, laboratory abnormalities occur only in a minority of patients (7.3%) with HDP but the rate increases with the severity of the disease.^27^ Of note, other investigations should be performed as the need arises such as the development of target organ dysfunction typical of PE with severe features. For instance, clotting profile (including international normalised ratio, fibrinogen and activated partial thromboplastin time) should be assessed in patients who develop evidence of thrombocytopenia or coagulopathy.

It is pertinent to draw further attention to the laboratory investigations used for monitoring already diagnosed cases of PE without severe feature, gestational hypertension and TGH. Serum creatinine helps with monitoring of renal function and levels above 120 mmol/L is an indication to consider delivery.^3^ Haemoglobin concentration is usually elevated because of volume depletion in PE^13^ but may be decreased if there is haemolysis. ALT is a good marker of hepatic disease^24^ although in PE-related hepatic dysfunction, AST is the initial transaminase preferentially released into peripheral circulation such that the circulatory concentration of AST dominates ALT (at least initially) and levels of these transaminases may be part of the evidence used to exclude other differential diagnosis of PE.^28^ Platelet count is aimed at detecting thrombocytopenia, which is a complication of PE but may be a part of criteria for diagnosing HELLP syndrome.

Concerning obstetric ultrasonography in gestational hypertension and PE without severe features, the NICE guidelines recommend once 2 weekly evaluation.^17^ The ACOG guidelines of June 2020 recommends that ultrasonography should be performed every week to assess amniotic fluid index and every 3–4 weeks to assess foetal growth.^28^ In low resource settings, the frequency of ultrasonography for foetal evaluation should be at least once every 2 weeks particularly in PE without severe features. The frequency should also not be longer than once every 2 weeks in gestational hypertension and TGH. If there is foetal growth restriction for instance, the severity including abnormalities in the umbilical and other foetal artery dopplers will determine the frequency of ultrasonography.^29^ Non-stress test should be performed at least once weekly. The second case in the present report demonstrates a lack of ready access to prenatal ultrasonography because of the high volume of patients waiting for the imaging and lack of hospital bed-space for inpatient care. As a result of socioeconomic challenges, the patient defaulted the scheduled appointment for ultrasonography and foetal demise occurred within 2 weeks following the diagnosis of PE. It also shows how cultural and socioeconomic challenges can interfere with postnatal care given that the second patient went home on day 2 postpartum against medical advice to perform traditional burial rite of her baby but did not return as planned and subsequently defaulted postnatal clinic visits because of the pressure from her workplace. These failings increase the risk of perinatal and maternal complications. Although the clinical issues are paramount, understanding the patients’ health beliefs is also important for carrying patients along during clinical encounters. The refusal of hospital treatment requires the clinicians to explore the patient’s agenda and negotiate any disparity. Patients’ behaviours and choices are often influenced by their perceptions and may not agree with the doctors’. Exploring the reason(s) for encounter therefore becomes critical. Unfortunately, the patient was not referred for further counselling by a social worker or clinical psychologist.^30^

Key take-home messages are shown in Table 1. Of note, the primary care providers help in preventing complications of HDP^31^ and the flow diagram shown in Figure 1 is a good guide that may assist with follow-up (intervals and the basic investigations to be performed at each visit to promote good outcomes). Further research on our recommendations is also suggested. Nonetheless, following arrival of a stable pregnant woman to a PHC, the following should be performed to diagnose or manage HDP: (1) measure the BP using a validated device and approved technique^1^; (2) use available tests such as dipstick to assess spot urine for proteinuria (3) provide health education on importance of antenatal care, self-awareness of symptoms of HDP and where possible the value of using validated home device to monitor BP^1^; (4) ascertain if there are symptoms or complaints and address them; (5) perform physical examination including cardiovascular and abdominal exam; (6) make diagnosis and risk categorise clients into low- or high-risk pregnancy with stable patients placed on prenatal vitamins including calcium whilst those at increased risk of HDP should also receive calcium and prophylactic aspirin starting early in the second trimester; (7) high-risk women should also receive calcium and emergency treatment where appropriate such as rapid-acting antihypertensive drug for severe hypertension and referred to a higher level of care^32,33,34^; (8) low-risk women should be managed and followed-up in the PHC clinic and or level 1 hospitals; (9) patients with pre-hypertension (BP 135–139/85–89 mmHg) should be followed-up within 3–7 days to repeat their BPs^3^ as they tend to develop hypertension (BP ≥ 140/90 mmHg) and are as well likely to have poor pregnancy outcomes including eclampsia^35^; (10) patients with HDP should be investigated and followed-up as illustrated in Figure 1. Additional algorithms on approach to HDP for the primary care physician is freely available online at https://safpj.co.za/index.php/safpj/article/view/5095/6009.^36^ To successfully implement our recommendations there has to be support and changes in the health policy, health system, levels of community involvement and accessibility to healthcare facilities. And the actions that may evolve before institutionalisation of the new recommendation are creation of awareness, commitment to implement, preparation to implement, implementation of the recommendations, integration of the new recommendation into routine practice and sustenance of the new practice. The details of actions required from different stakeholders to successfully implement the recommendations on HDP are contained in a table in the South African 2019 guidelines on HDP.^3^

**TABLE 1:** Key take-home messages.

Serial No.	Key points
1.	Transient gestational hypertension and pre-eclampsia increase the risk of adverse pregnancy outcomes.
2.	Socioeconomic challenges interfere with the management of hypertensive disorders of pregnancy.
3.	Stringent laboratory monitoring of a newly diagnosed new-onset hypertensive disorder of pregnancy should include at least testing blood levels of serum **C**reatinine, **H**aemoglobin concentration, **A**lanine transaminase and **P**latelets (**CHAP**) weekly.
4.	The use of recommendations in Figure 1, increase of in-hospital bed-spaces, ready access to obstetric ultrasonography and public health education on the value of antenatal clinic follow-up visits are important measures to improve pregnancy outcomes in hypertensive disorders of pregnancy.
5.	Robust studies are required to guide the frequency and types of routine laboratory testing in hypertensive disorders of pregnancy.

## Conclusion

The outcomes of HDP are often unpredictable and dramatic and the use of stringent recommendations in Figure 1 for monitoring the patients is valuable. However, an increase in the number of bed-spaces available in the hospital, ready access to obstetric ultrasonography and public health education on the value of antenatal clinic follow-up visits are important measures to improve pregnancy outcomes in HDP.
